# Adiponectin deficiency accelerates brain aging via mitochondria-associated neuroinflammation

**DOI:** 10.1186/s12979-023-00339-7

**Published:** 2023-04-01

**Authors:** Kaiwu He, Lulin Nie, Tahir Ali, Zizhen Liu, Weifen Li, Ruyan Gao, Zena Zhang, Jianjun Liu, Zhongliang Dai, Yongmei Xie, Zaijun Zhang, Gongping Liu, Ming Dong, Zhi-Jian Yu, Shupeng Li, Xifei Yang

**Affiliations:** 1 State Key Laboratory of Oncogenomics, School of Chemical Biology and Biotechnology, Peking University Shenzhen Graduate School, Shenzhen, 518055 China; 2 Shenzhen Key Laboratory of Modern Toxicology, Shenzhen Medical Key Discipline of Health Toxicology (2020-2024), Shenzhen Center for Disease Control and Prevention, Shenzhen, 518055 China; 3 Department of Anesthesiology, Shenzhen People’s Hospital, The Second Clinical Medical College, Jinan University, Shenzhen, 518020 Guangdong China; 4 Department of Anesthesiology, The First Affiliated Hospital, Southern University of Science and Technology, Shenzhen, 518020 Guangdong China; 5 Shenzhen Engineering Research Center of Anesthesiology, Shenzhen, 518020 Guangdong China; 6 State Key Laboratory of Biotherapy and Cancer Center, West China Hospital, Sichuan University and Collaborative Innovation Center of Biotherapy, Chengdu, 610041 China; 7 Institute of New Drug Research, College of Pharmacy, Jinan University, Guangzhou, 510632 China; 8 Department of Pathophysiology, School of Basic Medicine, Key Laboratory of Ministry of Education of China and Hubei Province for Neurological Disorders, Tongji Medical College, Huazhong University of Science and Technology, Wuhan, China; 9 Guangzhou International Bio Island, Guangzhou, 510005 Guangdong Province China; 10 Department of Infectious Diseases and Shenzhen Key Laboratory for Endogenous Infections, The 6Th Affiliated Hospital of Shenzhen University Health Science, Center. No 89, Taoyuan Road, Nanshan District, Shenzhen, 518052 China; 11 Campbell Research Institute, Centre for Addiction and Mental Health, Toronto, ON Canada; 12 Department of Psychiatry, University of Toronto, Toronto, ON Canada

**Keywords:** Adiponectin, Aging, HDAC1, Neuroinflammation, Mitochondria, BV2 Cells

## Abstract

**Background:**

A wide spectrum of changes occurs in the brain with age, from molecular to morphological aspects, and inflammation accompanied by mitochondria dysfunction is one of the significant factors associated with age. Adiponectin (APN), an essential adipokine in glucose and lipid metabolism, is involved in the aging; however, its role in brain aging has not been adequately explored. Here, we aimed to explore the relationship between APN deficiency and brain aging using multiple biochemical and pharmacological methods to probe APN in humans, KO mice, primary microglia, and BV2 cells.

**Results:**

We found that declining APN levels in aged human subjects correlated with dysregulated cytokine levels, while APN KO mice exhibited accelerated aging accompanied by learning and memory deficits, anxiety-like behaviors, neuroinflammation, and immunosenescence. APN-deficient mice displayed aggravated mitochondrial dysfunction and HDAC1 upregulation. In BV2 cells, the APN receptor agonist AdipoRon alleviated the mitochondrial deficits and aging markers induced by rotenone or antimycin A. HDAC1 antagonism by Compound 60 (Cpd 60) improved mitochondrial dysfunction and age-related inflammation, as validated in D-galactose-treated APN KO mice.

**Conclusion:**

These findings indicate that APN is a critical regulator of brain aging by preventing neuroinflammation associated with mitochondrial impairment via HDAC1 signaling.

**Supplementary Information:**

The online version contains supplementary material available at 10.1186/s12979-023-00339-7.

## Background

Aging is the unavoidable time-dependent decline of organ functionality leading to disease and death. As average life expectancy increases, the consequences and influences of advancing age become apparent and enhanced [1]. Therefore, research goals include understanding molecular mechanisms by which to modulate the aging process and promote healthy aging for prolonged life [2, 3].

Mitochondrial impairment is a key hallmark of aging [4, 5]. Neurons, with their high energy demands, are thus particularly sensitive to mitochondrial dysfunction. Oxidative stress generates reactive oxygen species (ROS), alters ATP production, and triggers inflammatory disorders involved in aging, indicating that mitochondrial defects are early key initiators of cellular perturbations that promote aging [6, 7]. Recent studies reveal that mitochondrial homeostasis and longevity are linked to epigenetic alterations of histone deacetylases (HDACs). In contrast, histone deacetylases promote gene expression in the mitochondrial stress response, immunity, and metabolism. HDAC thus has a significant role in the mitochondrial stress response and, thus, in longevity [8–10].

Besides their central role in energy metabolism, mitochondria are primary mediators of inflammation, and impaired mitochondria can modulate innate immunity via redox-signaling or direct inflammasome activation [11]. The adult brain must maintain a balance between pro- and anti-inflammatory cytokines; this balance shifts to proinflammatory cytokines with age, making the aged brain more vulnerable to stress and disease [12]. Increased cytokines, including IL-6, activated microglial cells, and inflammatory signaling like NF-kB, have been reported in the aged brain, indicating dysregulated mitochondria's role as the inflammatory process driver [13].

The aging process is also characterized by changes in body composition, such as the decline in growth hormones and insulin resistance [14]. Significant impairments of adipose lipogenesis, adipokines, and cytokine levels are associated with aging and contribute to the onset of age-related diseases [2, 14]. Adipose tissue deficiency and lipodystrophy are associated with dysregulated adipokines, which provide the basis for age-associated adverse metabolic consequences, including insulin resistance, hyperglycemia, and dyslipidemia, indicating that the adipose endocrine system is critically important for maintaining whole-body homeostasis [15]. Thus, metabolic regulators, including adipokines, are critical players in aging. Adiponectin (APN) is the most abundant adipocytokine primarily secreted by adipose tissues and regulates various physiological processes via its receptors (AdipoR1, AdipoR2, and T-cadherin) [16]. It has antioxidative and anti-inflammatory effects in multiple cells under different pathological conditions, apart from regulating glucose and lipid metabolism [17]. Previous studies have focused on the beneficial effects of APN in early life stages using young animal models; however, it remains to be explored the APN beneficial role in aging [18]. Moreover, APN deficiency has also been shown to increase neuroinflammation advancing with age and cognitive impairments [19, 20]. However, the molecular mechanisms underlying age-related neuroinflammation and adiponectin dysregulation have yet to be determined [21]. The present study explores the potential roles of APN and its association with mitochondrial impairment in aged humans and in APN knock-out (KO) vs. wild-type (WT) mice treated with D-galactose to speed brain aging cognitive decline [22]. These studies suggest that APN is a critical regulator of brain aging by preventing neuroinflammation associated with mitochondrial impairment via HDAC1 signaling.

## Results

### APN correlates with age, and its deficiency accelerated brain aging in mice

**Fig. 1:**
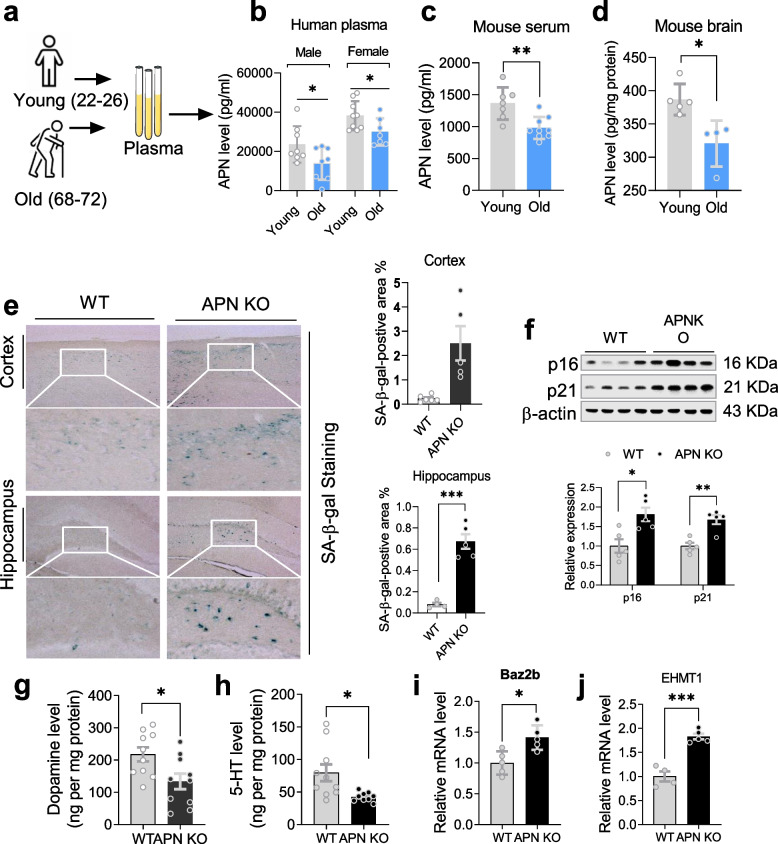
APN level declined in aging subjects, and its deficiency correlated with immunosenescence. **a-b**: APN level changes in the plasma of aged male/female human individuals (*n* = 7–9). **c-d**: Bar graphs representing APN level changes in the serum and cerebral cortex of young (6-month old) and aged (15.5-month old) APN KO mice (*n* = 4–9). **e**: SA-β-gal staining and quantification in the brain of aged mice (*n* = 5–6). **f**: Representative immunoblots and bar graphs showing expression of p16 and p21 in the cortex of aged mice (*n* = 5). **g-h**: Bar graphs showing dopamine and serotonin levels in the cortex of aged mice (*n* = 9–10). **i-j**: mRNA level of Baz2b and EHMT1 in the cortex of aged mice (*n* = 4–5). Data expressed as mean ± SEM, *p < 0.05, **p < 0.01, ***p < 0.001

Initially, we examined the correlation between APN and aging by measuring APN level changes in the aged subjects (humans/mice). APN declined in human plasma, murine serum, and brain tissue (Fig. 1a-d). To further validate the correlation between APN and aging, APN KO mice were employed to measure aging-associated senescence markers, including β-galactosidase, p16, and p21 [23]. Significantly higher expression of senescence-associated β-galactosidase (SA-β-gal) and p16 and p21 (hallmarks of senescence) were detected in the cerebral cortex and hippocampus of aged APN KO mice (Fig. 1e-f). Levels of dopamine (DA) and serotonin (5-HT) were significantly decreased (Fig. 1g-h), as previously noted in aging [24, 25], while Euchromatic Histone lysine Methyltransferase 1 (EHMT1) and Bromodomain Adjacent to Zinc finger domain 2B (Baz2b) expression was significantly increased (Fig. 1i-j). EHMT1 and Baz2b expression were measured to validate further these findings, which were deemed conserved epigenetic regulators preventing healthy aging [26].

### APN deficiency correlated with anxiety and cognitive impairment in mice in aged subjects

As age is accompanied by cognitive decline and deterioration of emotional function [19], we examined the anxiety-like behavior of APN KO vs. WT mice at 8 and 10 months of age. As shown in the figure (Supplemental Fig. 1), APN KO, relative to WT mice, spent more time in the center and open arm of the elevated plus-maze at 10 months than at 8 months. Also, at 13 months, WT and APN KO mice showed high freezing time during the training session in a fear conditioning test. In contrast, APN KO mice displayed reduced freezing time during contextual and cue-induced memory tasks (Supplemental Fig. 1), a measure of elevated cognitive dysfunction. Overall, our data strongly supported that APN deficiency could accelerate brain aging.

### APN level correlated with aging-related inflammation, and its deficiency enhanced neuroinflammation in mice

**Fig. 2:**
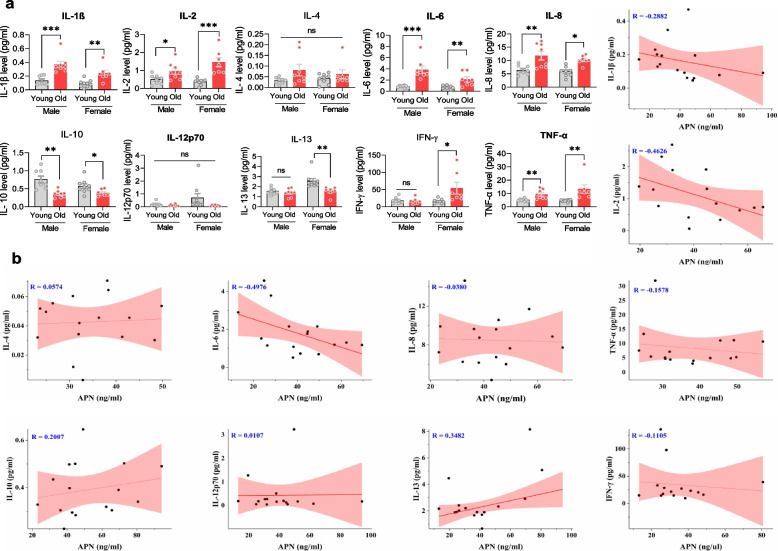
APN deficiency correlates with peripheral inflammation in aging subjects. **a**: Bar graphs showing APN level changes in plasma cytokine levels of young and old male/female human subjects (*n* = 7–9). **b**: Regression analysis, showing correlation of APN level with changes in cytokine levels. Data expressed as mean ± SEM, *p < 0.05, **p < 0.01, ***p < 0.001

Chronic inflammation is one of the essential characteristics of aging that is usually accompanied by increased proinflammatory indices [27]. This proposal was supported by significantly elevated levels of most such indices in aged humans (Fig. 2a). Comparatively, the imbalance between proinflammatory (IL-1β, IL-2, IL-6, IL-8, IFN-γ, TNF-α) and anti-inflammatory (IL-4, IL-10, IL-12p70, IL-13) cytokines was more evident in females. The correlation between APN levels and inflammatory cytokines in female samples was examined further. Notably, the APN level was negatively correlated with IL-2 and IL-6 but positively correlated with IL-13 (Fig. 2b), indicating the APN level was associated with an age-related inflammation disorder.

**Fig. 3:**
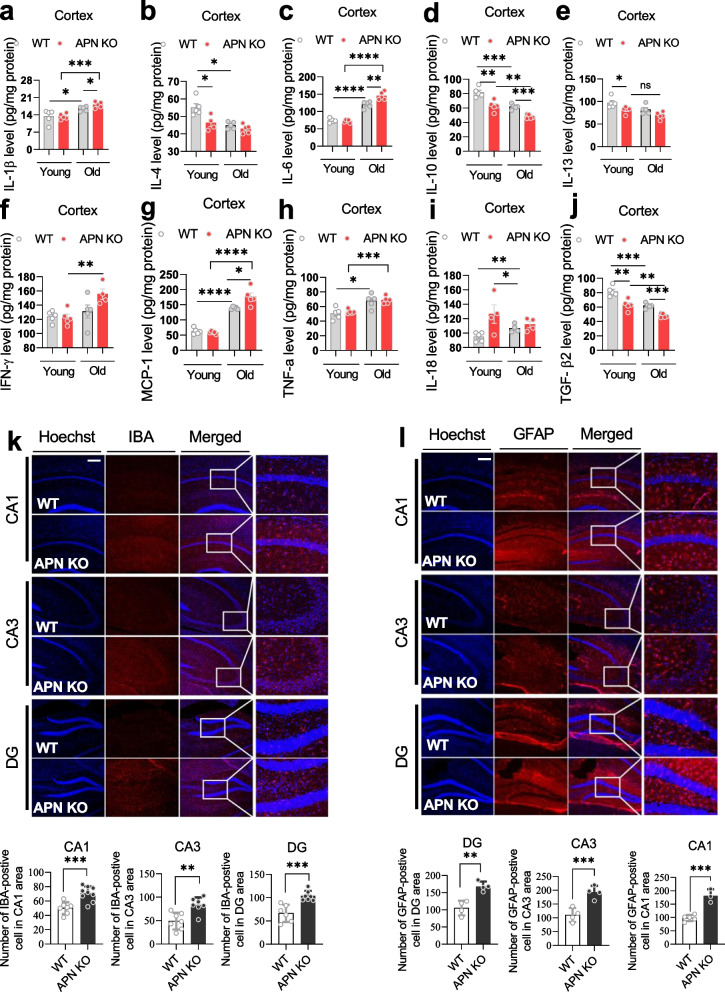
APN deficiency correlates with cytokine level and neuroinflammation in the APN KO mice. **a-j**: Cytokine level change in the cerebral cortex of APN KO mice (*n* = 4–6). **K-I**: Immunostaining (original magnification: × 200) and bar graphs show expression changes of IBA-1 and GFAP in the cortex of the experimental subjects (*n* = 4–6). Data expressed as mean ± SEM, *p < 0.05, **p < 0.01, ***p < 0.001, ****p < 0.0001. Scale bar, 100 μm

Next, we sought to determine the neuroinflammatory changes in the brains of aged APN KO mice. Initially, cytokines were measured in the cerebral cortex of APN KO and WT mice aged 6 months (young) and 15.5 months (old). Dysregulated levels of cytokines were detected in young mice, which were further accelerated with aging (Fig. 3a-j). A significantly increased level of some (such as IL-1β, IL-6, and MCP-1) but not all proinflammatory cytokines were found in the brains15.5-month-old-old APN KO mice compared with the age-matched WT mice. Both glial cellular markers (IBA-1/GFAP) were increased in the hippocampus of the APN KO mice, indicating that APN deficiency accelerated glial activation (Fig. 3k-l). Furthermore, there was significantly increased expression of NLRP3, NF-kB, and caspase 1. In contrast, expression of HO-1 and NRF2 were decreased (Supplemental Fig. 2), findings consistent with neuroinflammatory changes in the brain of APN KO mice. These changes indicated that APN deficiency shifted the balance of pro-and anti-inflammatory factors to create a more proinflammatory age-related state.

### APN deficiency enhanced mitochondrial impairment in aged mice

**Fig. 4:**
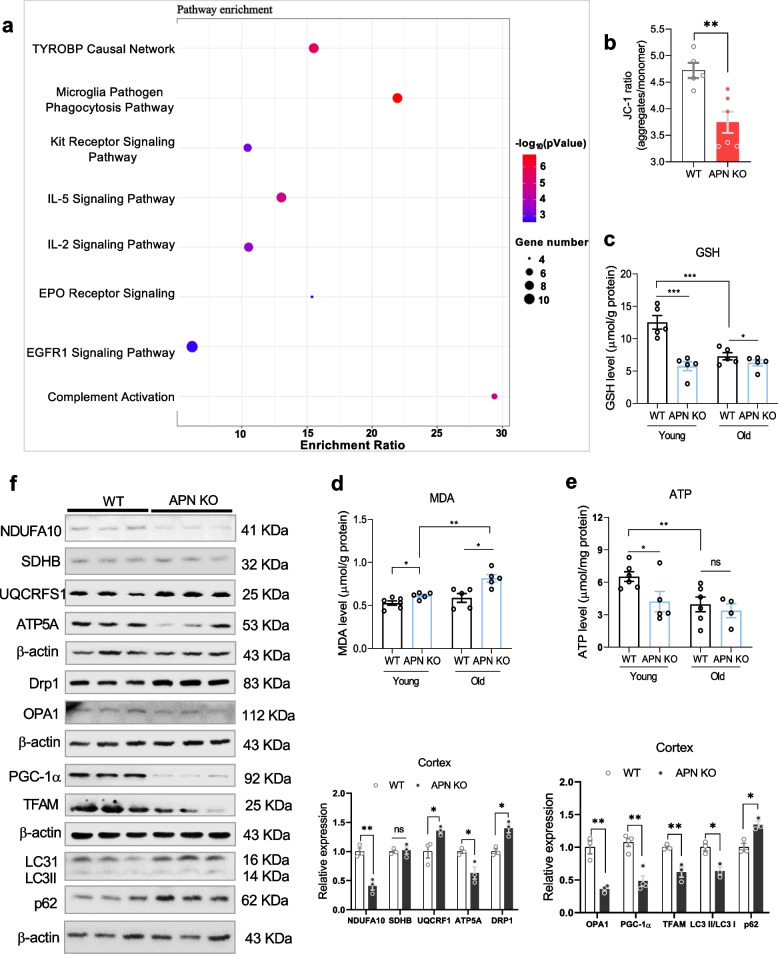
APN-deprived aged mice displayed mitochondrial impairments. **a**: TMT-labeled proteomics. **b**: JC-1 aggregation ratio (*n* = 4–6). **c-e**: Representative bar graphs showing relative levels of ATP, MDA, and GSH in the experimental subjects (*n* = 4–6). **f**: Immunoblots and their quantitative column graphs, showing expression of NDUFA10, SDHB, UQCRFS1, ATP5A, Drp1, OPA1, PGC-1α, TFAM, LC3BI/II, and p62. Immunoblot densities were optimized by standard (GAPDH/β-actin) (*n* = 3). Data expressed as mean ± SEM, *p < 0.05, **p < 0.01, ***p < 0.001

Mitochondrial impairment associated with oxidative stress and altered ATP levels is considered the critical hallmark of brain aging [28–30]. Mass spectrometry analysis identified a total of 5392 proteins, of which 399 proteins in the APN KO group were differentially expressed (Supplemental Table 4). The pathway analysis of these differentially expressed proteins showed enrichment in inflammatory signaling pathways, such as complement activation, IL-2 signaling and IL-5 signaling (Fig. 4a). Further, the JC-1 aggregation ratio was found to be significantly reduced in the APN KO mouse brain (Fig. 4b), showing that the mitochondrial membrane potential in APN KO mice is much lower than that of the normal control group. Further, a decreased level of ATP was found in the brain tissue of 6-month-old APN KO mice compared to age-matched WT controls. Moreover, the malonaldehyde (MDA) level was significantly increased, while glutathione (GSH) decreased in APN KO mice at 15.5 months compared with WT (Fig. 4c-e). These results indicated that APN deficiency could lead to mitochondrial impairment.

To validate the association between APN deficiency and mitochondrial impairment, we further investigated mitochondrial physiological markers in the cerebral cortex of 15.5-month WT vs. APN KO mice (Fig. 4f). NDUFA10 (Complex I) and ATP5A (Complex V) were significantly decreased in APN KO compared to WT mice, suggesting the involvement of APN deficiency in mitochondrial dysfunction. However, UQCRFS1 (Complex III) was significantly increased in APN KO mice, which might account for the significant increase in MDA. To further investigate mitochondrial dysfunction, we evaluated the expression of proteins involved in mitochondrial dynamics, mitochondrial biogenesis, and autophagy. Drp1 and p62 were significantly increased while OPA1, TFAM, PGC-1α, and LC3BII decreased in the cerebral cortex of aged APN KO vs. WT mice, suggesting that APN deficiency increased mitochondrial fission and decreased mitochondrial fusion, which may lead to mitochondrial fragmentation and neuronal degeneration.

### APN deficiency aggravates brain aging via HDAC1signaling

**Fig. 5:**
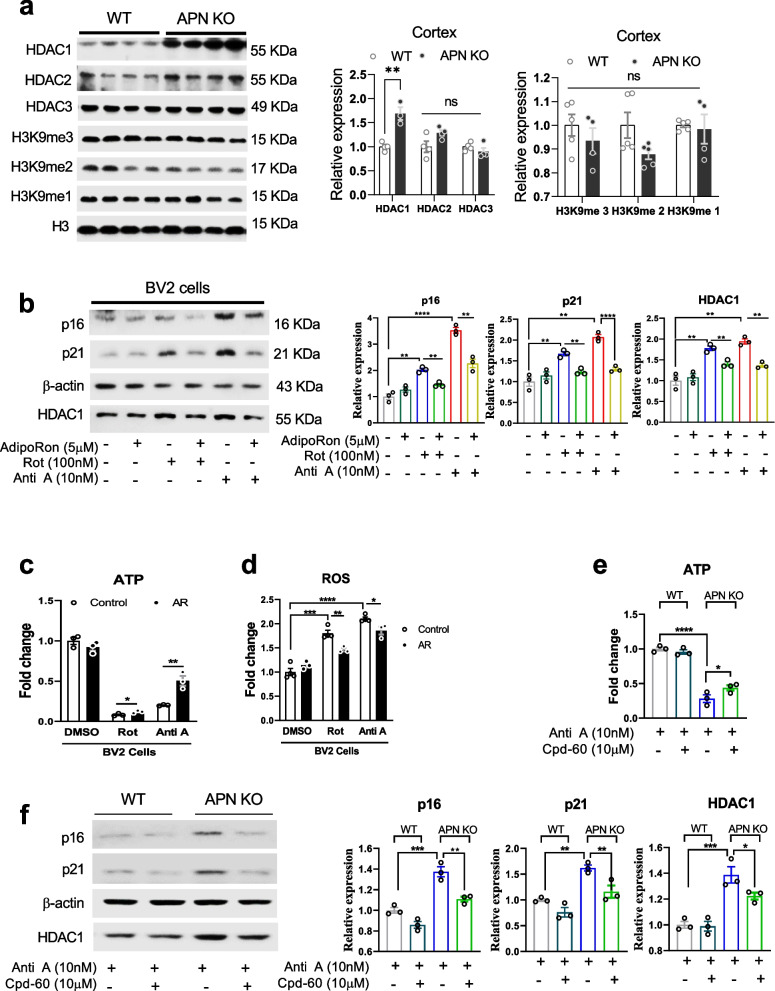
HDAC1 is associated with augmented aging in APN-deficient mice. **a**: Immunoblots and their quantitative column graphs, showing expression of HDAC1, 2, 3, H3K9me3, 2, and 1, in the cerebral cortex of age-matched APN KO vs. WT mice (*n* = 4–5). **b**: Representative immunoblots and bar graphs show p16, p21, and HDAC1 expression in AdipoRon-, Rot- and Anti A-treated BV2 cells (*n* = 3). **c-d**: Bar graphs showing the level of ATP and ROS in AdipoRon-, Rot- and Anti A-treated BV2 cells (*n* = 3). **e**: ATP level in the Anti A- and Cpd-50-treated primary microglial cells from WT and APN KO mice (*n* = 3). **f**: Representative immune blots and bar graphs showing relative expression of p16, p21, and HDAC1. Immunoblot densities were optimized by standard (GAPDH/β-actin) (*n* = 3). Data expressed as mean ± SEM, *p < 0.05, **p < 0.01, ***p < 0.001, ****p < 0.0001

Previous studies strongly support the role of HDACs (HDAC1/2) in the expression of UPRmt (UPR mitochondrial) genes [10]. We sought to determine whether mitochondrial impairment correlated with HDAC expression in APN KO conditions. Initially, the expression of HDACs, including HDAC1, HDAC2 and HDAC3, was examined in the cerebral cortex of APN KO vs. WT-aged mice (Fig. 5a). HDAC1 was markedly increased. In contrast, no significant changes were found for HDAC2 and HDAC3. No significant differences were found in the methylation level of H3K9me1/2/3 found (Fig. 5a).

**Fig. 6:**
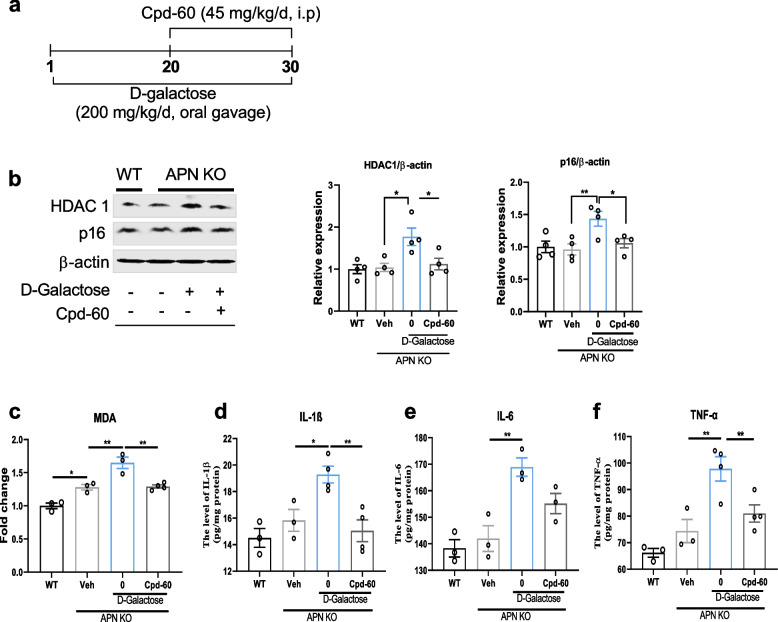
Cpd-60 reversed HDAC1-associated changes in D-galactose-treated APN KO mice. **a**: Experimental schedule/approach. **b**: Immunoblots and bar graphs show the expression of HDAC1 and p16 in the experimental murine cortex (*n* = 4). **c**, **d**, **e**, and **f**: Representative column showing fold changes in the MDA, IL-1β, IL-6, and TNF-α. Immunoblot densities were optimized by standard (GAPDH/β-actin) (*n* = 3–4). Data expressed as mean ± SEM, *p < 0.05, **p < 0.01

Next, we sought to determine the potential causal relation between HDAC1 with age and mitochondrial impairment. BV2 cells were treated with Rotenone (Rot) and Antimycin A (Anti A) to induce mitochondrial dysfunction and cell senescence [23]. BV2 cells are microglia cells derived from c57/BL6 murine, which retains microglial morphological and functional characteristics. Rot and Anti A significantly enhanced p16, p21, and HDAC1 expression in the BV2 cells. However, most of these changes could be reversed by AdipoRon (AR) treatment (Fig. 5b). ATP and ROS changes were also checked to validate mitochondrial dysfunction, which was also decreased by AR treatment (Fig. 5c-d). Next, primary microglial cells (PMCs) from the WT and APN KO mice were treated with an HDAC1 inhibitor (Cpd-60) to evaluate the role of HDAC1 in Anti A-induced mitochondrial dysfunction and cell senescence. As shown in Fig. 5e, the ATP level was significantly decreased in PMCs from APN KO mice compared with that of WT mice, and Cpd-60 treatment enhanced mitochondrial function in PMCs from APN KO mice. Consistently, Cpd-60 treatment significantly decreased p16, p21, and HDAC1 expression in Anti A treated PMCs isolated from APN KO mice (Fig. 5f). To further examine the roles of HDAC1 in aging-related pathological processes, 8-month-old APN KO mice were treated by gavage with D-galactose for 30 days and, between days 20 and 30, with the HDAC1 inhibitor (Cpd-60) administered by intraperitoneal injection (Fig. 6a). D-galactose significantly enhanced HDAC1 and p16 expression, which was reduced by Cpd-60 treatment (Fig. 6b), validating the causal relation of HDAC1 in APN deficiency-related aging and senescence. Additionally, increased levels of MDA and cytokines (IL-1b, IL-6, and TNF-α) were found in D-galactose-treated APN KO vs. WT mice, which was also reversed by HDAC1 antagonism (Fig. 6c-f). These findings indicated that APN deficiency elicited the aging process in mouse brains via HDAC1 signaling. Besides, given that adiponectin receptors are important in regulating glucose and lipid metabolism, aging could decrease basic metabolism. We further explore the effect of Cpd-60 treatment on the expression of AdipoR1 and AdipoR2 in D-galactose-induced aged BV2 cells. Unfortunately, the results showed that Cpd-60 did not significantly alter the expression of AdipoR1 and AdipoR2 (Supplemental Fig. 3). The potential underlying mechanisms of Cpd-60 treatment alleviated brain aging are certainly worth further investigation.

## Discussion

Our findings demonstrated that the murine brain APN level is negatively correlated with the advance of biological age. APN deficiency accelerated brain aging and increased anxiety and cognitive impairment. Furthermore, it aggravates mitochondrial impairment via HDAC1 signaling.

Our results show that dysregulated HDAC1, p16 and p21 and mitochondria-associated gene expression in the aged APN KO mice further support the interplay between APN and aging. However, the deficits could be reversed by the HDAC1 inhibitor Cpd 60 both in vivo and in vitro, validating that HDAC1-mediated mitochondrial dysfunction may underlie the APN-deficient to facilitate the aging process. In addition, we found neuroinflammation in APN KO-aged mice, as demonstrated by glial cell activation and elevated proinflammatory cytokines. Interestingly, these cytokine abnormalities are closely correlated with APN changes in the age human population.

Age-related immune response dysfunction may lead to chronic inflammation followed by immunosenescence [31, 32]. In agreement with previous reports, we found abnormal cytokine levels in the plasma of the aged human sample, further validated by increased senescence markers. Systemic inflammation could lead to neuroinflammation, as the peripheral cytokine impairment may parallel neuroinflammation [31, 33, 34]. Our results showed that APN deprivation accelerated proinflammatory cytokines, followed by brain aging. It indicates a critical association between aging and neuroinflammation. As an initial defensive mechanism, the aged brain responds to stimuli (stress) by producing an exaggerated cytokine level from glial cell activation, leading to severe detriments, including prolonged sickness and cognitive impairment [12, 32]. For instance, glial cells have biological properties that might impact learning and memory. Microglia can remove synapses in an activity-dependent manner altering neural networks, while astrocytes can modulate synaptic transmission and may couple multiple neurons and synapses into functional assemblies [35, 36]. Besides, the present study found that APN deficiency caused significant enrichment in some neuroinflammation-related pathways, such as microglia pathogen phagocytosis pathway and complement activation, highlighting the involvement of neuroinflammation in APN deficiency aggravating brain aging. Simultaneously, further studies are merited to illuminate the homeostatic imbalance of inflammation in the aged brain of APN-deficient subjects.

Published studies indicate that aging cells exhibit enhanced mitochondrial DNA mutation and functional impairments in response to persistent oxidative stress [37, 38]. Moreover, imbalanced ROS production due to damaged mtDNA stimulates inflammasome (danger-sensing multiprotein platform) formation [39, 40]. It shows an interplay between mitochondrial impairment and neuroinflammation, as evidenced by close interactions between increased inflammation and altered mitochondrial function [41]. Thus, potential inflammation-suppressive factors, including adiponectin, could reduce mitochondrial damage and thus regulate inflammation. Following our previous reports [42], APN KO-aged mice displayed microglial cell activation together with IL-6 and TNF-α production in the hippocampal tissues of the mouse brain. In the present study, we found dysregulated neuroinflammation and peripheral inflammation in aged APN KO mice, demonstrating a key role of the APN-linked inflammatory response in brain aging. Notably, altered neuroinflammation was accompanied by mitochondrial impairment and senescence, further validated by mitochondria-associated inflammatory pathway impairments shown by mass spectrometry analysis and JC-1 ratio calculation. The exact mechanisms whereby glial activation is induced in APN KO mouse brain require clarification. As suggested by the present results and those of augmented mitochondria in APN deficiency conditions may trigger the increased secretion of proinflammatory cytokines leading to a neuroinflammatory response [43].

Histone deacetylases (HDACs) modify epigenetic activities and have been recently linked to mitochondrial homeostasis and longevity [10, 44]. Similarly, we have previously reported the role of HDAC1 in lipopolysaccharide-induced neuroinflammation and depression models [45]. APN deficiency enhanced neuroinflammation, aggravated mitochondrial dysfunction, and increased memory impairment, indicating that HDAC can contribute to cognitive impairment via neuroinflammation-associated mitochondrial dysfunction. However, this phenomenon has not been linked to aging or APN-associated activities and expression. Our results show that HDAC1 upregulation in aged APN KO mice is accompanied by enhanced neuroinflammation and senescence, which indicates a causal link between HDAC1 and mitochondrial dysfunction in APN-deprived conditions. Similarly, the HDAC1 level was significantly increased in the APN KO mice upon D-galactose administration, whereas the HDAC1-inhibitor reversed brain aging by decreasing mitochondrial deficits and p16, MDA, and cytokine levels.

## Conclusion

In conclusion, this study revealed that APN level decline could be associated with brain aging via exaggerated neuroinflammation and cognitive impairment. Concurrently, neuroinflammation coincides with mitochondrial impairments via HDAC1 signaling in the APN-deprived condition. Hence these findings explore a crucial interplay between APN deficiency and aging and may provide HDAC1 as a therapeutic target for mitochondrial defects in aging.

## Methods

### Mice and human

APN KO mice (B6;129-Adipoq^tm1Chan^/J, Stock No: 008195) were purchased from the Jackson Laboratory (Maine, USA). The WT mice (C57BL/6 J) were purchased from Weitong Lihua Limited Company (Beijing, China). All mice were housed in a pathogen-free facility with a 12 h light–dark cycle (lights on at 6:00 am, lights off at 6:00 pm) with ad libitum access to food and water. According to the previous method, D-Galactose (200 mg/kg/d, oral gavage) was used to induce the animal model of accelerated aging [22]*.* The Animal Care and Use Committee of the Experimental Animal Center at Shenzhen Center for Disease Control and Prevention approved the animal experiments. The approval number for the animal experiment is 2019038. Further, we followed the already published protocol [46] for animal/group selection for a research study.

Informed consent for all human samples was obtained following the Ethical Committee of Shenzhen Center for Disease Control and Prevention guidelines (Ethical committee Approval number: R2018020). Young individuals (*n* = 17) and BMI-matched older individuals (*n* = 15) have additional characteristics, as shown in Supplemental Table 1. As demonstrated by %HbA1c, all individuals were normoglycemic. The exclusion criteria included: smoking, antibiotic usage, auto-immune disease, diabetes mellitus, hypertension, pregnancy, and neurodegenerative diseases.

#### SA-β-gal for frozen sections

Detailed analyses are mentioned in the Supplementary data.

#### Human plasma cytokine assays

Detailed analyses are mentioned in the Supplementary data.

#### Mitochondrial function and oxidative damage

The detailed assay was mentioned in the Supplementary data.

#### Proteomics analysis

We performed a proteomics study as previously reported [45]. Detailed analyses are mentioned in the Supplementary data.

#### Cell culture

We performed a proteomics study as previously reported [23, 47]. Detailed analyses are mentioned in the Supplementary data and Supplementary tables 2 and 3.

#### Flow cytometry, ELISA, RT-PCR, Western blot, and Immunofluorescence

Detailed analyses are mentioned in the Supplementary data and Supplementary tables 2 and 3.

### Behaviors analysis

Detailed analyses are mentioned in the Supplementary data.

### Statistical analysis

Data are presented as the mean ± SEM and analyzed using GraphPad Prism 8.0 statistical software (GraphPad Software, Inc., La Jolla, CA, USA). A two-tailed unpaired Student's test was applied to compare two groups statistically. Simultaneously, One-way analysis of variance (ANOVA) was employed to determine the statistical significance of differences among groups and follow Dunnett's multiple comparison test. A probability value of p^*^ < 0.05, p^**^ < 0.01, p^***^ < 0.001 and p^****^ < 0.0001 was considered statistically significant.

## Supplementary Information

**Additional file 1.**

**Additional file 2.**

**Additional file 3.**

**Additional file 4.**

## Data Availability

All data generated or analyzed during this study are included in this published article [and its supplementary information files].

## Abbreviations

HDACs: Histone deacetylases
mtDNA: Mitochondrial DNA
APN: Adiponectin
AdipoR1: Adiponectin receptor 1
AdipoR2: Adiponectin receptor 2
AR: AdipoRon
ROS: Reactive oxygen species
Cpd 60: Compound 60
DA: Dopamine,
5-HT: Serotonin
MDA: Malonaldehyde
GSH: Glutathione
UPRmt: UPR mitochondrial
Rot: Rotenone
Anti A: Antimycin A
PMCs: Primary microglial cells
