# Supplementary material for: Adiponectin deficiency accelerates brain aging via mitochondria-associated neuroinflammation
Source: Immun Ageing. 2023 Apr 1;20:15. doi: 10.1186/s12979-023-00339-7 (PMC10067304; doi:10.1186/s12979-023-00339-7)
Supplement: Supplementary file 1 — Additional file 1. [file 12979_2023_339_MOESM1_ESM.docx]

**Figure S1: APN KO aged-mice displayed anxiety and cognitive impairment**

**a:** Bar graphs show total distance and time in the center of open field test, time in the open arm of elevated plus-maze test in 8-month-old APN KO vs. WT mice. **b:** Total distance traveled and time in center of open field test, and time in open arm spent of elevated plus-maze test in 10-month-old APN KO vs. WT mice. **c:** Bar graphs showing freezing time during training and test of fear conditioning (at 13-months of age) of APN KO vs. WT mice. Data expressed as mean ± SEM, **p* < 0.05, ***p* < 0.01, *****p* < 0.0001.

**Figure S2: APN deficiency increases the expression of neuroinflammatory markers**

**a:** Representative images of immunoblots showing NRF2, HO-1, NLRP3, Caspase 1, and NF-kB**. b, c, d, e and f:** Bar graphs showing relative expression of Nrf2, HO-1, NLRP3, Caspase 1 and NF-kB, respectively. Immunoblot densities were optimized by standard (GAPDH/β-actin). Data expressed as mean ± SEM, **p* < 0.05, ***p* < 0.01, ****p* < 0.001.

**Figure S3: The effect of HDAC1 inhibitor (Cpd-60) treatment on the expression of Adiponectin receptors in D-galactose-induced aged BV2 cells.**

a-b: Representative immunoblots and bar graphs showing expression of AdipoR1 and AdipoR2 in D-galactose-induced aged BV2 cells with or without Cpd-60 treatment. Data expressed as mean ± SEM.
