# Supplementary material for: Adiponectin deficiency accelerates brain aging via mitochondria-associated neuroinflammation
Source: Immun Ageing. 2023 Apr 1;20:15. doi: 10.1186/s12979-023-00339-7 (PMC10067304; doi:10.1186/s12979-023-00339-7)
Supplement: Supplementary file 2 — Additional file 2. [file 12979_2023_339_MOESM2_ESM.docx]

**Table S1: Baseline characteristics of Human individuals**

|  | **Young** | **Old** |
| --- | --- | --- |
| Total N | 17 | 15 |
| Age, years [mean (rang)] | 24.1 (22-27) | 72.5 (68-78) |
| Body weight, kg [mean (rang)] | 58.1 (39.4-88.6) | 65.8 (38-89.7) |
| HbA1c, % [mean (rang)] | 5.1 (4.8-5.4) | 5.6 (5.1-6.0) |
| BMI, kg/m2 [mean (rang)] | 21.68 (18.6-30.3) | 24.51 (15.82-29.80) |
| Males [N (%)] | 8 (47.06%) | 8 (53.33%) |
| Females [N (%)] | 9 (52.94%) | 7 (46.67%) |

**Table S2: The primary antibodies used in this study.**

| **Antibody** | **Cat.** | **Type** | **Dilution** | **Source** |
| --- | --- | --- | --- | --- |
| p16 | sc-1661 | Mouse | 1:500 | Santa cruz |
| p21 | ab109199 | Rabbit | 1:1000 | Abcam |
| NRF2 | ab92946 | Rabbit | 1:1000 | Abcam |
| HO-1 | 43966S | Rabbit | 1:1000 | Cell signaling technology |
| NLRP3 | ab263899 | Rabbit | 1:1000 | Abcam |
| Caspase 1 | 22915-1-AP | Rabbit | 1:1000 | Proteintech |
| NFκB | ab32360 | Rabbit | 1:1000 | Abcam |
| NDUFA10 | ab103026 | Rabbit | 1:1000 | Abcam |
| SDHB | ab14714 | Mouse | 1:1000 | Abcam |
| UQCRFS1 | ab131152 | Rabbit | 1:1000 | Abcam |
| ATP5A | ab14748 | Mouse | 1:1000 | Abcam |
| Drp1 | sc-271583 | Mouse | 1:500 | Santa cruz |
| OPA1 | sc-393296 | Mouse | 1:500 | Santa cruz |
| PGC-1α | ab54481 | Rabbit | 1:1000 | Abcam |
| TFAM | ab47517 | Rabbit | 1:1000 | Abcam |
| LC3B | 2775S | Rabbit | 1:1000 | Cell signaling technology |
| P62 | ab109012 | Rabbit | 1:1000 | Abcam |
| HDAC1 | 34589S | Rabbit | 1:1000 | Cell signaling technology |
| HDAC2 | 57156S | Rabbit | 1:1000 | Cell signaling technology |
| HDAC3 | 85057S | Rabbit | 1:1000 | Cell signaling technology |
| H3K9me1 | ab9045 | Rabbit | 1:1000 | Abcam |
| H3K9me2 | ab115159 | Rabbit | 1:1000 | Abcam |
| H3K9me3 | ab8898 | Rabbit | 1:1000 | Abcam |
| H3 | ab1791 | Rabbit | 1:3000 | Abcam |
| β-actin | sc-47778 | Mouse | 1:3000 | Santa cruz |

**Table S3: The main chemical reagents, cell mediums, ELISA kits and other reagents used in this study.**

| **Reagents** | **Cat.** | **Source** |
| --- | --- | --- |
| DCFH-DA | D6883 | Sigma |
| rotenone | HY-B1756 | MCE |
| antimycin A | HY-100558 | MCE |
| AdipoRon | S872570 | Macklin |
| Compound 60 | HY-100719 | MCE |
| D-Galactose | D810319 | Macklin |
| ATP assay kit | S0026B | Beyotime |
| JC-1 assay kit | C2003S | Beyotime |
| MDA assay kit | S0131S | Beyotime |
| mitochondrial extraction kit | C3606 | Beyotime |
| GSH assay kit | A006-2-1 | Nanjing Jiancheng Bioengineering Institute |
| DA assay kit | H170 | Nanjing Jiancheng Bioengineering Institute |
| 5-HT assay kit | H104-1-1 | Nanjing Jiancheng Bioengineering Institute |
| β-galactosidase staining kit | K320-250 | BioVision |
| QuicKey Human ADP/Acrp30 | E-TSEL-H0020 | Elascience |
| Mouse ADP/Acrp30 | E-EL-M0002c | Elascience |
| Human plasma cytokine assays kit | LX-K15049D-X | LabEx |
| Mouse IL-1β ELISA Kit | E-EL-M0037c | Elascience |
| Mouse IL-4 ELISA Kit | E-EL-M0043c | Elascience |
| Mouse IL-6 ELISA Kit | E-EL-M0044c | Elascience |
| Mouse IL-10 ELISA Kit | E-EL-M0046c | Elascience |
| Mouse IL-13 ELISA Kit | E-EL-M0727c | Elascience |
| Mouse IFN-γ ELISA Kit | E-EL-M0048c | Elascience |
| Mouse MCP-1 ELISA Kit | E-EL-M3001 | Elascience |
| Mouse TNF-α ELISA Kit | E-EL-M0049c | Elascience |
| Mouse IL-18 ELISA Kit | E-EL-M0730c | Elascience |
| Mouse TGF-β2 ELISA Kit | E-EL-M1191c | Elascience |
| DMEM/F12 medium | C11330500BT | Gibco |
| Trypsin/Lys-C Mix | V5073 | Promega |
| TMT labeled kit | 90406 | Thermo |
