# Supplementary material for: Adiponectin deficiency accelerates brain aging via mitochondria-associated neuroinflammation
Source: Immun Ageing. 2023 Apr 1;20:15. doi: 10.1186/s12979-023-00339-7 (PMC10067304; doi:10.1186/s12979-023-00339-7)
Supplement: Supplementary file 4 — Additional file 4. [file 12979_2023_339_MOESM4_ESM.docx]

**Supplementary data**

**Mouse Behavioral Assays**

Open field and elevated plus-maze tests were used to assess anxiety-like behavior in mice. A fear conditioning test was used to assess cognitive impairment.

***Open field test***

The open field apparatus consisted of a plexiglas box (50 x 50 x 40 cm). The bottom of this apparatus was divided into 16 equal squares. The inner 25 x 25 cm area was defined as central; the remaining regions were defined as peripheral areas. Mice were allowed to explore the device for 5 min. The device was cleaned with 75% ethanol and allowed to dry completely between trials. The total distance traveled and the time spent in the center were recorded by Xeye software wwill will Shanghai, China).

***Elevated-plus maze test***

The elevated plus-maze comprised two open and two closed arms extending from a central platform. Each mouse was individually placed in the center area and allowed to explore the device for 5 min. The time and movement of each animal were recorded by Xeye software (Bio will, Shanghai, China). The time spent in the open arms was calculated to assess anxiety-like behavior.

***Fear conditioning test***

Fear conditioning was performed as previously described (21). The apparatus consisted of an acrylic chamber (25 × 25 × 25 cm) equipped with a stainless-steel grid floor. On the training day, each mouse was first allowed to explore the chamber for 6 minutes without any other behaviorally relevant stimulus. After that, mice received 3 paired presentations of a 30 s, 4 kHz, 80 dB auditory cue (CS) co-terminating with a 2 s, 0.5 mA scrambled footshock (US), followed by the addition of 2 min of free exploration without tone or shock stimuli. Each inter-trial interval was 2 min. The chamber was cleaned with 75% ethanol between sessions to avoid residue from the previous session. On the second day, mice were placed into the original chamber to assess contextual memory. Animals could explore the chamber for 8 minutes without a tone or shock stimulus. The cued memory was tested in a novel context on the third day with different odorants and shapes in the chamber. After a brief baseline period with no tone, the 80 dB tone sounded for 30 s at trial timepoints 120 s, 270 s, and 420 s. Then mice received the addition of 2 min of free exploration without any tone. AThe animalmovement was recorded and analyzed using a video-tracking system Bio will, Shanghai, China)

**Flow Cytometry**

Flow cytometry was used to measure the intracellular ROS level. In brief, the aged BV2 cells with or without AdipoRon treatment were incubated using 10μM 2',7’-dichlorofluorescein diacetate (DCFH-DA, Sigma, USA) for 30 min at 37℃. Then cells were washed 3 times with PBS and collected for analysis through BD Accuri C6 Plus (BD, Bioscience, USA).

**Western blot**

Protein samples from animals or cells were extracted with RIPA lysis solution containing protease and phosphatase inhibitor (Thermo, USA) and then separated by SDS-PAGE and transferred to a PVDF membrane. The membrane was blocked with 5% nonfat milk for 1 h at room temperature and then incubated with primary and secondary antibodies. The primary antibodies used in this study are listed in Supplementary Table 2. The protein level was measured using the Pierce^TM^ ECL Western Blotting Substrate kit (Thermo, USA) and quantified using ImageJ software.

**ELISA**

The levels of human plasma APN, mouse APN from serum and brain tissue, and inflammatory cytokines including IL-1β, IL-6, TNF-α, MCP-1, IFN-γ, IL-18, IL-4, IL-10, IL-13, and TGF-β2 were measured using ELISA kits from Elabscience (Wuhan, China) according to the manufacture's protocol. Dopamine signaling, including dopamine (DA) and serotonin (5-HT), was detected using ELISA kits from the Nanjing Jiancheng Bioengineering Institute, China.

**RT-PCR**

Total RNA was extracted using TRIZOL reagent (Invitrogen, Germany) from brain tissues of 15.5 month-old WT and APN KO mice. RNA integrity and concentration were verified using Nanodrop2000 (Thermo, USA). PrimeScript RT-PCR Kit (TAKARA, Japan) was used to perform reverse transcription to synthesize cDNA. Gene expression was quantified following the instructions provided with SYBR PremixEx Taq^TM^ (TAKARA, Japan). The β-actin gene was selected as a housekeeping reference. The following primers were used for quantification: EHMT1 (F) GGC ACC TTT GTC TGC GAA TAC and (R) AGA ACC GAG CGT CAA TGC AG; Baz2b (F) GCT CTA GAC GTC AGG CTT GTT and (R) TTC ACA CCG CTG GTC TTG TT; β-actin (F) TCC GGC TCA GAA CTA CAG TGT AAT and (R) TGC GGC GTT TTC ATG GT. The RT-PCR procedure was performed under the following amplification conditions: pre-denaturation at 95℃ for 2 min; 40 cycles including denaturation at 95℃ for 5 s, annealing at 54℃ for 3,0 s, and extension at 70℃ for 34 s. Finally, the 2^-ΔΔCt^ method was used to analyze gene expression.

**Immunofluorescence**

Paraffin-embedded sections were deparaffinized with dimethyl benzene and rehydrated in graded alcohol solutions, followed by a citric acid antigen-repair buffer to unmask epitopes. After washing 3 times with PBS, the sections were blocked with blocking buffer (0.3% Triton X-100 + 3% bovine serum albumin in PBS) for 60 min, followed by overnight incubation at 4℃ with primary antibodies, including mouse monoclonal anti-GFAP and rabbit polyclonal anti-Iba1. Sections were labeled with fluorescent secondary antibodies as follows: Alexa Fluor 488 goat anti-mouse IgG (H+L) and Alexa Fluor 568 goat anti-rabbit IgG (H+L). DAPI (4, 6-diamidino-2-phenylindole) was used to counterstain the nuclei. The images were acquired with a confocal microscope (Leica DMI6000, Germany) under a 200× objective and analyzed using ImageJ software.

**SA-β-gal for frozen sections**

WT and APN KO mice were deeply anesthetized with 4% chloral hydrate and perfused via the left ventricle with PBS to remove intravascular blood. The brains were rapidly removed from the skull and divided into two mid-sagittal halves. The left hemi-brains were fixed in 4% paraformaldehyde for 2 days, dehydrated, embedded in paraffin, and 5-µm sections prepared for immunofluorescence staining. The right hemi-brains were used to prepare frozen sections. For SA-β-gal staining, the right hemi-brains were fixed with 4% paraformaldehyde (PFA) for 2 days and dehydrated through a sucrose gradient (10% sucrose, 1 day; 20% sucrose, 1 day; 30% sucrose, 2 days). After that, 20-µm frozen sections were cut and rehydrated 3 times with PBS in a 6-well plate. SA-β-gal staining was performed using a kit (BestBio, China). Briefly, sections were immersed in a fixation solution provided with the kit for 15 min and subsequently washed with PBS 3 times. 2 ml of working solution of β-galactosidase with X-gal was placed in each well, and the plate was maintained at 37℃ for 48 h. SA-β-gal-positive areas were quantified by counting stained and unstained areas and expressing the results as percent of SA-β-gal-positive areas over the total area.

**Cell culture**

The mouse microglia cell line (BV2) was purchased from the Cell Bank of the Chinese Academy of Sciences (Beijing, China). The BV2 cells were cultured in 6-well plates with DMEM/F12 supplemented with 10% FBS and incubated at 37℃ in an atmosphere of 5% CO_2_. For senescence induction, BV2 cells were treated with 100 nM rotenone (Rot) or 10 nM antimycin A (Anti A) for 5 days (19). Aged BV2 cells were treated with 5μM of the common APN receptor agonist AdipoRon.

Primary microglia culture werculturesoped using a published method (20). Briefly, brain tissue from postnatal (day 1-3) APN KO mice and WT mice was isolated, cut into tiny pieces, and digested with trypsin for 15 min. Dissociated tissues mixed with glia were then plated into T-25 culture flasks containing DMEM/F12 with 10% fetal bovine serum, GlutaMAX (Invitrogen), and 1% penicillin/streptomycin. After culture in a 5% CO_2_/37℃ incubator for 14 days, the flasks were shaken at 220 rpm for 4 h at 37℃ to harvest the primary microglia. After that, the microglia were plated in 6-well plates at a density of 5 x 10^5^ cells per well to induce senescence and 10μM Cpd-60 treatment.

**Human plasma cytokine assays**

Human plasma cytokine levels of IL-1β, IL-2, IL-6, IL-8, IFN-γ, TNF-α, IL-4, IL-10, IL-12p70, and IL-13 were measured by Meso Scale Discovery (MSD) according to the manufacturer’s protocol. Briefly, plasma samples were added to the plate and incubated at room temperature. After 2 h incubation, the plate was washed 3 times before the addition of detection antibodies and read using a MESO QuickPlex SQ120. The data were analyzed using MSD Workbench software v.4.0.

**Mitochondrial function and oxidative damage**

*Assessment of ATP level*

Mitochondrial function was assessed by measuring ATP production using a commercially available kit (Beyotime, China) according to the manufacturer's protocol. In principle, The bioluminescence assay is based on the reaction of ATP with recombinant firefly luciferase and its

substract luciferin. Luciferase catalyzes the formation of light from ATP and luciferin. It is the emitted light that is linearly related to the ATP concentration, which is measured with a luminometer. ATP levels were measured by using a standard curve method.

*Assessment of lipid peroxidation*

The lipid peroxidation was determined using an MDA assay kit (Beyotime, China). Briefly, samples were washed with PBS and lysed with lysis buffer. After centrifuge, the supernatant was collected and used for MDA detection. MDA level was measured following the manufacturer's protocol. A microplate reader (Tecan, Austria) was used to measure the absorbance of cellular MDA at 532 nm. Protein concentrations were determined by BCA protein assay kit.

*Assessment of GSH level*

The GSH level was determined using the GSH assay kit (Nanjing Jiancheng Bioengineering Institute, China). According to the manufacture’s method, samples were homogenized with PBS, and centrifuge at 2500 rpm at 4℃ for 15 min. The supernatant was collected, and the absorbance was assessed at 405 nm.

*Mitochondrial membrane potential*

To evaluate the effect of APN deficiency on mitochondrial function, the change of mitochondrial membrane potential was detected by JC-1. Briefly, mitochondria were firstly extracted from brain tissue of 15.5-month old WT and APN KO mice via using a mitochondrial extraction kit (Beyotime, China) according to the manufactures recommendations. The 100 μg mitochondria were incubated with 0.9 ml JC-1 working solution for 20 min at 37℃. Because JC-1 is a green-fluorescent monomer at low membrane potential, with the membrane potential of energized mitochondria promoting the formation of red-fluorescent J-aggregates. Thus, the level of mitochondrial membrane potential (MMP) is expressed as the relative ratio of red (J-aggregates) and green (monomer) fluorescence.

**Proteomic analysis**

***Protein preparation and labeling***

Mice hippocampal tissue samples from 6-month old WT and APN KO mice were lysed with 8 M urea containing protease inhibitor cocktail (1X), centrifuged at 4℃, 14000 g for 30 min, followed by collected into a new 1.5 ml centrifuge tube. Then, a total of 100 μg of protein from each sample were incubated with 10 mM DTT at 55℃ for 60 min, followed by 25 mM IAA treatment for 60 min at room temperature. Each fully denatured sample was incubated with 4 μg Trypsin/Lys-C Mix at 37℃ for 3 h in a final concentration of 8 M urea buffer. Then above reaction was diluted with PBS to reduce urea concentration to 1M, followed by incubating overnight at 37℃. The digested peptides from each sample were desalted, dried, and finally labeled with TMT 10plex reagents.

***Peptide Fractionation***

TMT-labeled samples were fractionated using nanoflow DIONEX UltiMate 3000 RSLCnano System (Thermo Fisher Scientific, USA) coupled with C18 resin (300 Å, 5 μm; Varian, Lexington, MA). and a silica capillary column (75 μm ID, 150 mm length; Upchurch, Oak Harbor, WA). The samples were separated by a gradient from 5% to 90% ACN at a flow rate of 0.30 μl/min. Peptides were separated into 45 fractions, which were consolidated into 15 fractions. The fractions were subsequently dried and re-dissolved in 0.1% formic acid.

***LC-MS/MS and database searching***

LC-MS/MS analyzed the re-dissolved peptides with the same LC system equipped with Q Exactive HF-X Quadrupole-Orbitrap (Thermo Fisher Scientific, USA) in data-dependent acquisition (DDA) mode. A single full-scan mass spectrum obtained the data acquisition in Orbitrap (350–1800 m/z, 70,000 resolution) followed by the top 20 data-dependent MS/MS scans.

The resultant mass spectrometric data were analyzed using Proteome Discoverer 2.6 (Thermo Fisher Scientific, USA) using a protein database composed of the Mus musculus fasta database downloaded from UniProtKB on 26 Aug 2020, containing 17053 reviewed protein sequences. The enzymes to trypsin were set with two missed cleavage tolerance. The static modifications were then fixed to carbamidomethylation (+57.021464) of methionine and acetylation (+42.010565) of peptides' N-termini. Precursor ion mass tolerance was set to 20 ppm. Similarly, the fragment ion mass tolerance was also selected as 20 mmu for all MS/MS spectra obtained. Quantitative precision was expressed with protein ratio variability. Differentially expressed proteins (DEPs) were expressed as fold change at 1.2 and 0.83, respectively.

***Bioinformatic analysis***

Pathway enrichment was performed via online WEB-based Gene SeT AnaLysis Toolkit (http://www.webgestalt.org/option.php), followed by RStudio 4.0.0 for visualization.
